# Predictors of Speech-in-Noise Understanding in a Population of Occupationally Noise-Exposed Individuals

**DOI:** 10.3390/biology13060416

**Published:** 2024-06-05

**Authors:** Guillaume Andéol, Nihaad Paraouty, Fabrice Giraudet, Nicolas Wallaert, Vincent Isnard, Annie Moulin, Clara Suied

**Affiliations:** 1 Institut de Recherche Biomédicale des Armées, 1 Place Valérie André, 91220 Brétigny sur Orge, France; vincent.isnard@def.gouv.fr (V.I.); clara.suied@def.gouv.fr (C.S.); 2 iAudiogram—My Medical Assistant SAS, 51100 Reims, France; paraouty@iaudiogram.com (N.P.); wallaert@iaudiogram.com (N.W.); 3 Department of Neurosensory Biophysics, INSERM U1107 NEURO-DOL, School of Medecine, Université Clermont Auvergne, 63000 Clermont-Ferrand, France; fabrice.giraudet@uca.fr; 4 Laboratoire des Systèmes Perceptifs, UMR CNRS 8248, Département d’Etudes Cognitives, Ecole Normale Supérieure, Université Paris Sciences et Lettres (PSL), 75005 Paris, France; 5 Department of Otorhinolaryngology-Head and Neck Surgery, Rennes University Hospital, 35000 Rennes, France; 6 Centre de Recherche en Neurosciences de Lyon, CRNL Inserm U1028—CNRS UMR5292—UCBLyon1, Perception Attention Memory Team, Bâtiment 452 B, 95 Bd Pinel, 69675 Bron Cedex, France; annie.moulin@cnrs.fr

**Keywords:** extended high frequency, speech in noise, amplitude modulation detection, frequency modulation detection, distortion products of otoacoustic emissions, electrocochleography, hearing questionnaire

## Abstract

**Simple Summary:**

In professional environments, communication errors can facilitate the occurrence of accidents. Professionals working in noisy environments may have difficulty understanding speech in noise. This may be due to the masking effect of noise, but also due to auditory lesions caused by regular exposure to noise. The current audiologic tools in occupational medicine are insufficient to both assess difficulties in understanding speech in noise and monitor workers’ hearing. The aim of this study was to evaluate the relationships between different variables thought to relate to speech in noise understanding, and to identify the most important variables. Hearing thresholds at 12,500 Hz, a frequency higher than those measured with conventional audiometry, were found to be strongly related to the ability to understand speech in noise. Regular monitoring of such extended high-frequency audiometry could therefore make it possible to offer appropriate care before auditory function deteriorates critically.

**Abstract:**

Understanding speech in noise is particularly difficult for individuals occupationally exposed to noise due to a mix of noise-induced auditory lesions and the energetic masking of speech signals. For years, the monitoring of conventional audiometric thresholds has been the usual method to check and preserve auditory function. Recently, suprathreshold deficits, notably, difficulties in understanding speech in noise, has pointed out the need for new monitoring tools. The present study aims to identify the most important variables that predict speech in noise understanding in order to suggest a new method of hearing status monitoring. Physiological (distortion products of otoacoustic emissions, electrocochleography) and behavioral (amplitude and frequency modulation detection thresholds, conventional and extended high-frequency audiometric thresholds) variables were collected in a population of individuals presenting a relatively homogeneous occupational noise exposure. Those variables were used as predictors in a statistical model (random forest) to predict the scores of three different speech-in-noise tests and a self-report of speech-in-noise ability. The extended high-frequency threshold appears to be the best predictor and therefore an interesting candidate for a new way of monitoring noise-exposed professionals.

## 1. Introduction

Noise is a polymorphous concept. Usually, it can refer to at least three definitions. Firstly, it may correspond to sound stimulation that is irrelevant to the listener, masking a signal of interest and thus diminishing its intelligibility. Secondly, noise can also be considered as a physical aggressor inducing damage to the auditory system. Finally, noise is an environmental stressor that can, for example, disrupt sleep and disturb the cardiovascular system. In the present study, we will mainly use the first two definitions (masker and aggressor). Understanding speech in a noisy background is difficult. It is particularly difficult for individuals occupationally exposed to noise as a physical aggressor, e.g., professional motorcycle drivers. The difficulty in understanding speech arises because the background noise contains energy in the same frequency regions as the speech (energetic masking [1]). It also arises because prolonged or excessive noise exposure can alter the auditory periphery structures, and may ultimately lead to noise-induced hearing loss [2]. However, individuals chronically exposed to noise, even with normal or near-normal audiometric thresholds, can exhibit difficulties understanding speech in noise [3,4]. 

Recently, several research groups have explored the hypothesis that noise exposure can induce selective synaptic loss at the synapses between the inner hair cells (IHCs) and the low-spontaneous-rate auditory nerve fibers in the cochlea, often occurring with otherwise normal or near-to-normal audiograms (see the seminal paper by Kujawa and Liberman [5]; for a review, see [6,7]. This synaptopathy has also been called hidden hearing loss (term coined by Schaette and McAlpine [8]), because its effect is not revealed by conventional audiometric measures. It is now widely assumed that clinical measures that are more sensitive than the conventional audiogram are needed [9]. However, a gold standard of these new tests and best practices is still to be defined [9,10], in order to detect early signs of hearing deficits, and to implement better prevention programs. 

These new tests should be defined in relation to the difficulties in understanding speech in noise. The evaluation of speech-in-noise performance in humans varies along a large variety of factors (type of target speech, type of masker, type of response, signal-to-noise ratio, and type of paradigms, to name a few), each providing different insights into an individual’s ability to process speech in noisy environments (review in [2]. An interesting way to differentiate them in the context of this study is by their lexical complexity (phoneme or syllable, words, and sentences). It has indeed been shown that this is one of the key factors for understanding the relative influence of the cognitive processes underlying and correlated to speech-in-noise tasks (review in [11]. Classically, tests with phonemes are less sensitive to cognitive factors than sentence recognition tests. To understand the apparent discrepancy in the literature regarding the existence of noise-induced cochlear synaptopathy in humans, DiNino et al. [12] showed that the choice of the target speech and the speech-in-noise task greatly impact on whether a relationship between the speech-in-noise performance and the assumed physiological proxies of synaptopathy (electrocochleography/auditory brainstem response wave I, middle ear muscle reflex) are observed. For instance, the tests with a low lexical complexity and which maximize the importance of fine temporal details were more likely to be correlated with proxy measures of synaptopathy in humans. 

The list of statistically significant predictors of speech-in-noise performance is vast, especially for individuals exposed to noise. A systematic overview being largely beyond the scope of this paper, here, we chose to focus instead on measures that would, in fine, be easily implemented in a prevention program—in addition to the conventional pure tone audiogram. Behaviorally, a decline in auditory temporal abilities (e.g., amplitude and frequency modulation thresholds) has been linked to a decline in speech-in-noise performance [13,14,15,16,17]. There is also now ample evidence for an association between extended high-frequency (EHF) audiometry, defined as frequencies above 8 kHz, and speech perception difficulties (review in [18]. Interestingly, here, noise exposure has been identified as one of the causes of EHF hearing loss [19,20]. To complement behavioral audiometric measures, electrophysiological measures of the cochlear function can be performed in individuals with normal hearing thresholds, and compared with the speech-in-noise performance (e.g., [21,22,23]). These tests include the measurement of the cochlear amplification function via the measurement of distortion product otoacoustic emission (DPOAE), or the synaptic activity between the IHC and the auditory nerve via the electrocochleography (EcochG) measurements. As explained before for speech-in-noise tasks, the different and sometimes opposite results in the literature regarding the existence and the measurement of noise-induced cochlear synaptopathy can also be linked to the very heterogenous methods used (see the recent reviews [24,25,26]). This discrepancy in the literature could also highlight the fact that variability in what we call normal thresholds or near-normal thresholds can potentially account for some of the so-called synaptopathy effects. In some of the studies in which noise exposure seems responsible for functional speech-in-noise differences in the absence of hearing loss, there is, of course, the possibility that differences in thresholds within the normal range can contribute nonetheless to the differences observed in the speech-in-noise performance [2,7]. In addition, cochlear synaptopathy is indeed very hard to study in humans, and is generally mixed with other outer hair cell (OHC) dysfunctions [22]. 

Finally, when studying noise-exposed individuals, the definition itself and the measurement of what is called “exposure” is crucial. As pointed out by Parker [22], one of the differences potentially explaining the discrepancies between noise-induced cochlear synaptopathy studies lies in the way noise exposure is measured. When noise exposure measurement is based on self-reports, no link is found between proxy of cochlear synaptopathy and speech-in-noise performance [20,27,28]. When controlled and homogeneous groups of individuals exposed to noise are studied (young and professional musicians in [29]; firearms users in [30]; train drivers in [31]), a correlation was found. Moreover, to investigate the effect of noise exposure, very often, groups of noise-exposed vs. controls are compared. This could be in contradiction with the idea that the outcome of noise exposure is possibly on a continuum from non-synaptopathy to synaptopathy with damage [9].

In the current study, we do not question the influence of different predictors on speech-in-noise performance, nor test the influence of one predictor on another (although it would be possible with this set of data). Instead, we investigate how to quantify and classify the various predictors of speech-in-noise performance in terms of importance. This approach has a direct clinical outcome as it allows us to establish which predictors are urgently needed for regular testing [2] in order to enhance existing hearing loss prevention policies. 

In fact, the choice of the statistical model and analysis is key to our study design as they influence the way we think about the design, as well as the conclusions we can draw from the data. To illustrate this point, Yeend et al. [32] recognized that one of the limitations of their study was the use of multiple comparisons, potentially resulting in falsely identified positive effects. More recently, Balan et al. [33] emphasized, with appropriate machine learning techniques, the importance of EHF audiograms in predicting speech-in-noise performance. 

In this paper, we use random forests—a machine learning tool for classification and regression. The random forest tool is intuitive, and, more importantly, it has an inherent capacity to produce measures of “variable importance”. Kim et al. [34] highlighted the usefulness of the random forest model, compared to other machine learning techniques, for predicting speech discrimination scores from pure tone audiometry thresholds. 

In this study, we investigated the relative importance of several audiometric, auditory, physiological predictors on the speech-in-noise performance. The speech-in-noise performance was assessed with three different speech-in-noise tests, with different degrees of lexical complexity (consonant identification; word in noise recognition; French sentence matrix test). Our listener group consists of individuals exposed to occupational noise: professional motorcyclists. This allowed us to have a homogeneous subject group and an easy proxy measure of noise exposure (number of years of motorcycling). All the participants had normal hearing thresholds (pure tone average (PTA): mean of thresholds at 500, 1000, 2000, and 4000 Hz, inferior to 20 dB HL) according to the reference of the International Bureau for Audiophonologie [35]. For all participants, we measured the EHF audiometry at 12.5 kHz; DPOAE to evaluate the OHC function; and EcochG to assess the auditory nerve function. Temporal processing was assessed using amplitude (AM) and frequency modulation (FM) detection thresholds. Finally, we evaluated the subjective auditory consequences of each subject’s noise exposure via the speech-in-noise pragmatic scale of the Speech, Spatial and Qualities of Hearing Scale (SSQ) questionnaire [36,37].

## 2. Materials and Methods

### 2.1. Overview

The experiment was conducted over a week during a span of 3 half-days, each dedicated to specific sets of experimental sessions. Two half-days were composed of a speech-in-noise audiometry test and a behavioral test (for instance, a session of consonants identification followed by a session of AM detection). The third one was composed of a speech-in-noise audiometry test, recordings of DPOAE and EcochG, and the questionnaires (demographic and SSQ speech-in-noise pragmatic scale). The order of all tests was randomized with at least one speech-in-noise test during each half-day.

### 2.2. Participants

**Figure 1:**
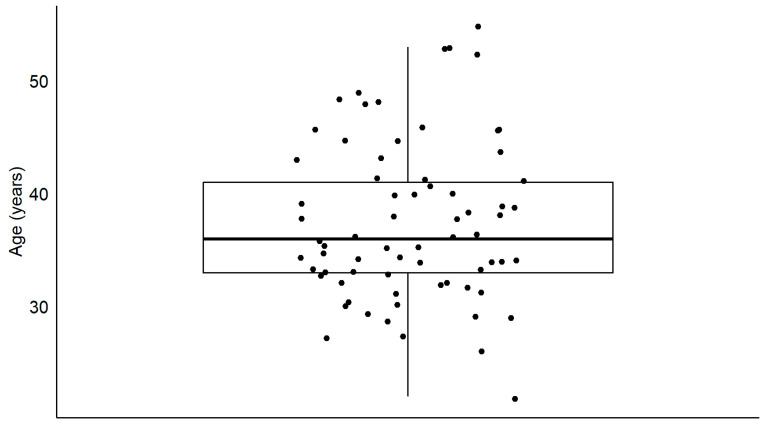
Distribution of the age of participants. The boxplots show the medial (horizontal bar) and the interquartile range (box). The whiskers reach from the lowest to the highest observed value within 1.5 times the interquartile range. Each dot shows the age of one participant.

Seventy-three participants (72 men; mean and standard deviation age: 38 ± 7.6 years (Figure 1) took part in the present study. All were professional motorcyclists occupationally exposed to noise (duration of exposure ranging between 1 year and 31 years, with a median duration of 8 years). The noise to which motorcyclists are exposed comes either from radio communications, air turbulence, or the motorcycle engine. The noise levels usually vary between 94.6 dB Leq8h and 103.6 dB. However, motorcyclists use noise-cancelling earplugs. Therefore, the actual levels vary between 69.6 and 81 dB Leq8h. In a typical week of work, the duration of daily noise exposure varies between 2 and 7 h. The median duration is approximately 4 h.

Three participants were excluded because their PTA was above or equal to 20 dB HL. The 70 remaining participants had a PTA considered normal in both ears according to the Internation Bureau for Audiophonology calculation [5]. Maximal age was limited to 55 years to reduce the risk of presbycusis. Informed consent was obtained from all participants involved in this study. This study was approved by Comité de protection des personnes sud-ouest et outre-mer II (IDRCB 2017-A00859–44).

### 2.3. Mobile Laboratory

**Figure 2:**
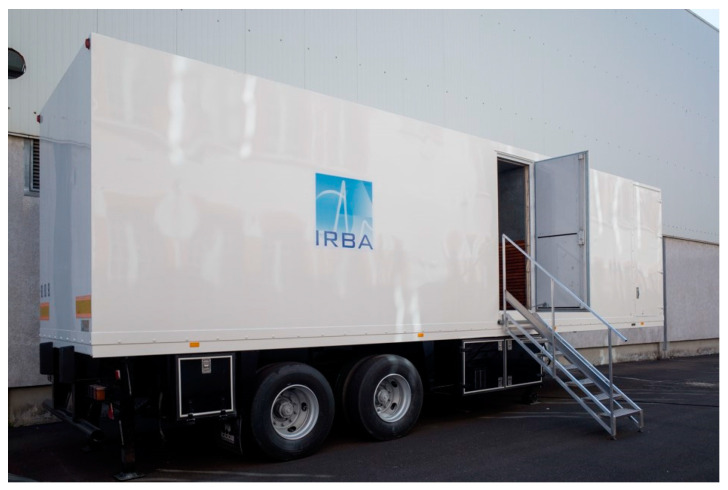
Exterior view of the mobile hearing laboratory.

**Figure 3:**
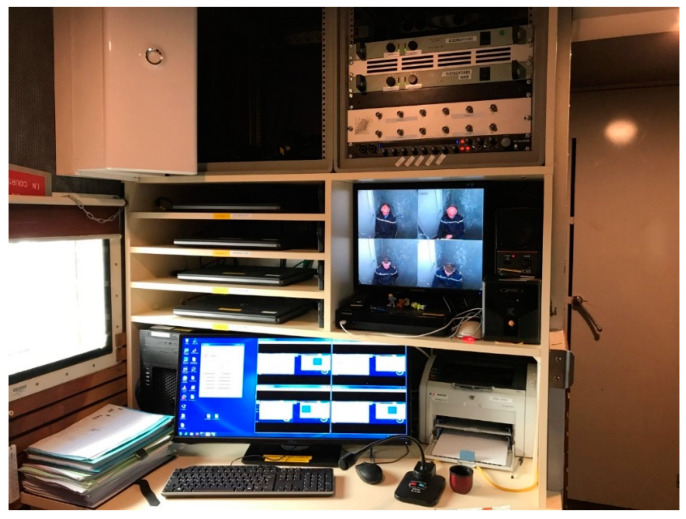
Interior view of the mobile hearing laboratory. In the center right of the setup, a video screen displays images of participants situated in the four booths. Positioned in the center left are four portable “follower” computers equipped with fold-down screens, to which the screens, keyboards, and mice of each booth are connected. Beneath these computers, there is the “leader” computer, positioned at the bottom center, with its screen visible. Additionally, the screens corresponding to the “follower” computers are also visible.

The tests were carried out in a mobile hearing laboratory (Figure 2 and Figure 3), consisting of four audiometric booths. Each booth is equipped with experimental instruments that can be remotely controlled from the control room. The four booths were used simultaneously to optimize the experimental time for a group of participants. An audio and video system enabled communication between the experimenter and each of the four participants individually or simultaneously, and was used to remind them of the instructions, maintain motivation, and monitor their state of arousal.

### 2.4. Speech-in-Noise Audiometry 

Each participant performed three different speech-in-noise audiometry tests: 1. the consonant identification test, 2. the word recognition test, and 3. the French matrix test (FrMatrix). The masking provided by the noise was energetic (no informational masking was used). A closed-set paradigm was used for the consonant identification test whereas an open-set paradigm was used for the word recognition and the FrMatrix tests. 

### 2.5. Consonant Identification Test

The consonant identification test consists of the presentation of 48 nonsense vowel–consonant–vowel–consonant–vowels (VCVCVs) spoken by a French female talker presented in a spectro-temporally modulated noise at −10 dB SNR. The signal was presented monaurally on the right ear. The 48 presentations came from three recordings of 16 French consonants (C = /p, t, k, b, d, g, f, s, ∫, v, z, ᶚ, l, m, n, r/), systematically associated with the vowel /a/. The duration of each presentation was, on average, 1272 ± 113 ms. 

For each trial, the participant had to indicate the perceived consonant by clicking on a matrix of 16 different consonants presented visually in front of them. No feedback was provided. The identification score corresponded to the percentage of correct answers. The presentation level was 65 dB SPL.

### 2.6. Words-in-Noise Recognition

Ten different lists were presented in the right ear of each participant. Each list consisted of 25 monosyllabic French words. Four different SNR ratios were compared: −5, 0, 5, 10 dB, in a speech-shaped noise and in silence. For each SNR, two lists (i.e., 50 words) were presented. The order of presentations was randomized and the association between lists and SNR conditions was counterbalanced. Each participant had to write, using the keyboard, the word they heard. Participants were instructed to write the words as they heard them, even if only one phoneme was heard, and to respect phoneme to grapheme conversion in the French language, not minding spelling mistakes. Each correspondence between the written word and target word from the list was then manually checked by 2 independent observers.

### 2.7. French Sentence Matrix Test

The French version of the sentence matrix test [38] was used to determine the speech reception threshold of the participants. The sentences are all constructed using the same pattern: a first name, a verb, a number, the name of an object and a color (for instance, “Jean Luc ramène trois vélos roses”). There are 10 possible choices for each word category. A test session consists of 20 sentences presented with an adaptative staircase procedure. The listener is seated one meter away facing the loudspeaker emitting the sentences and the noise. The participant’s task is to repeat aloud the words. The signal-to-noise ratio (fixed noise level at 65 dB SPL) varies from sentence to sentence depending on the number of correct words given by the participant in order to obtain the 50% speech reception threshold (SRT). Each participant performed three sessions. The final SRT of each participant (i.e., the dependent variable) is the best (i.e., lowest) value from the three sessions. The normative value is of −6 dB SNR (standard deviation 0.6 dB [39]).

### 2.8. Speech, Spatial and Quality of Hearing Questionnaire

In addition to the above-described behavioral measures of speech intelligibility in noise, the participant also completed a self-report measure. 

The Speech, Spatial and Qualities of Hearing Scale (SSQ) questionnaire enables measurement of a participant’s ability in various listening situations [36] using a numerical gradation from 0 (no, not at all) to 10 (yes, perfectly). The questionnaire is divided into three subscales: speech comprehension (14 questions), spatial hearing (17 questions), and hearing quality (18 questions).

The closer the numerical value is to 10, the more the subject feels able to perform the task described. We used a French version of the questionnaire that was previously validated [37,40]. The averages of items 1, 4, and 6 of the speech comprehension subscale were combined into the “speech-in-noise” pragmatic scale [41]. 

### 2.9. Predictors of Speech-in-Noise Tests

In order to identify the best predictors of speech intelligibility in noise, several physiological and behavioral measurements were conducted. Altogether, when taking into account several markers (i.e., variables) for each measure type, a set of 48 variables was obtained. They are all described below. They were all expected to predict to a certain degree the speech-in-noise performance as measured by the four speech-in-noise tests described above (consonant identification, word recognition, FrMatrix, and the speech-in-noise pragmatic scale of the SSQ). 

#### 2.9.1. Pure Tone Audiometry

**Figure 4:**
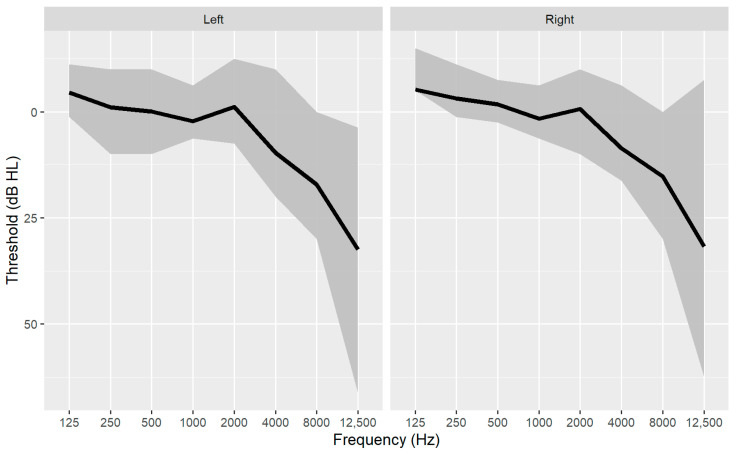
Audiometric thresholds as a function of frequency for left and right ear (N = 70). The black line shows the median, the gray area shows the interquartile range.

The audiometric thresholds were recorded with an automatic procedure with the EDM-Echodia Elios^®^ system (Le Mazet-Saint-Voy, France) with Radiohear headphones DD45 at the left ear and at the right ear for the frequencies 125, 250, 500, 1000, 2000, 4000, 8000, and 12,500 Hz (Figure 4). The 12,500 Hz frequency was defined as the EHF threshold. The four frequencies’ pure tone averages (PTA; 500, 1000, 2000, 4000) were computed in both ears, and the best ear PTA was identified as the lower PTA across the two ears. Therefore, nineteen predictor values were obtained from the pure tone audiometry.

#### 2.9.2. Amplitude and Frequency Modulation Detection Thresholds

A total of eight thresholds were obtained for each participant using a two-interval forced-choice procedure from the combination of modulation type (AM or FM), sinusoidal carrier signal frequency (500 or 4000 Hz), and stimulus intensity (10 or 60 dB SL). The standard signal was unmodulated, i.e., the modulation depth (Δ) was set to 0. The target signal was modulated and the value of the modulation depth, Δ, was adaptively modified in order to determine the threshold. All stimuli were generated digitally using a sampling rate of 44.1 kHz, and presented to participants at a presentation level of 10 dB SL or 60 dB SL, using Beyer DT 770 headphones and an external AudioEngine D3 sound card. Stimuli were presented monaurally to the right ear. Each trial consisted of a target modulated signal and a standard unmodulated signal, presented in random order, and separated by a 600 ms silence interval. The participant was instructed to indicate the stimulus containing the modulation, and was informed of the accuracy of their response by a light signal (green if correct, otherwise, red). Each stimulus’ duration was 1200 ms. 

Threshold was determined using a “2-down-1-up” method: Δf decreased when the participant responded correctly twice consecutively, and increased in the event of an error. The test stopped after 14 inversions, defined as an increase followed by a decrease in ∆ or vice versa. The detection threshold was calculated from the average ∆ over the last six inversions. Three threshold estimates were made for each intensity level condition (10 dB SL and 60 dB SL), and each type of modulation (AM and FM), and each carrier frequency (500 and 4000 Hz). The final value for each condition tested corresponded to the best performance obtained.

In our study, it appeared that some participants were not able to perform the task for three conditions in the FM detection task: 4000 Hz carrier frequency at 10 dBSL; 4000 Hz carrier frequency at 60 dB SL; and 500 Hz carrier frequency at 10 dB SL. To take this into account, we created 3 two-level categorical variables according to the ability of the participant to perform the task (able/not able). Therefore, 11 predictors were obtained from the AM and FM detection thresholds.

#### 2.9.3. Distortion Products of Otoacoustic Emissions

Distortion product otoacoustic emissions (DPOAEs) were collected with the EDM-Echodia Elios^®^ system (Le Mazet-Saint-Voy, France). An f2/f1 ratio of 1.20 was used at intensity levels of f1 = 75 dB SPL and f2 = 65 dB SPL. The amplitude of DPOAEs were recorded at frequencies of 1, 2, 3, 4, and 5 kHz in both ears (recorded values of −10 dB SPL or lower were discarded) to obtain 10 predictors per participant.

#### 2.9.4. Electrocochleography

Extratympanic electrocochleography (EcochG) was conducted with the Echodia Elios^®^ system (France). Two electro-encephalogram electrodes were placed on the forehead of the participant (one centered and one off-center, the two on the hairline). The extratympanic electrode was a gold-coated soft Tiprode, positioned in the outer ear canal. The electric impedances of the electrodes were checked to be below 5 kΩ. Acoustic stimuli were short clicks delivered at a rate of 11/s. The recordings were collected at 90 dB nHL, then at 80 dB nHL. For each level, the procedure consisted of averaging 500 responses, repeated two or three times depending on the consistency of the waveforms across the 500 responses. For each waveform, the amplitude of wave I was assessed by the difference in voltage between the first peak occurring between 1 and 2.5 ms and the next trough. Then, the amplitudes of the two most consistent waveforms were averaged. Furthermore, the slope of the input/output function obtained by linking the two stimulation levels (80 and 90 dB nHL) and the wave I amplitude was computed for each ear. Accordingly, six predictors (wave I amplitude at 80 and 90 dB nHL at both ears plus wave I slope at both ears) per participant were obtained.

#### 2.9.5. Random Forest Analysis

In addition to the 48 predictor variables described above, 3 were added: the age; the number of years of motorcycling; and the history of hearing pathology (otitis media or acute acoustic trauma). A total of 47 variables were continuous and 4 were categorical. The main goal here was to identify the most important predictors of speech-in-noise performance. 

In order to perform this importance analysis, we used random forest algorithms. Recently, biomedical research in general has found an interest in this machine learning paradigm, given its interpretability, its nonparametric approach with large use case, and the potential mix between continuous and categorical variables [42]. Random forests have already been identified as an interesting choice among machine learning algorithms in hearing sciences [33,34,43].

A random forest is a combination of 500 decision trees. Each decision tree is built from a random sample of the population and a random sample of the variables to reduce the risk of overfitting. Next, all 500 trees are combined to build a model and make a prediction. A prediction error is computed from the data excluded from the random samples (“error out of the box”). The difference between the observed value to predict and the actual prediction is represented by the mean square error (MSE). To assess the importance of a variable, the impact of random permutations of that variable is measured on the MSE. The more the MSE increases, the more important the variable is. 

In order to have a reliable measure of each variable’s importance, the non-scaled importance measure was computed on 10 subsamples and then averaged across samples. The subsamples were built by randomly selecting 75% of the original data sample. 

We used the *randomForest* R package Version: 4.7-1.1 with the hyperparameters set by default. Missing values were handled by the command “na.action==na.roughfix”.

In addition to the importance graph for each speech-in-noise test, we described the 9 most important variables and the correlations between the speech-in-noise performance and the variable (predictor). Hence, nine scatterplot graphs are plotted for each speech-in-noise audiometry test. On each scatterplot, the Spearman coefficient of correlation, its *p*-value, and the size sample are indicated. The Spearman coefficient was chosen against the Pearson coefficient because several variables were not normally distributed (e.g., speech-in-noise pragmatic scale, years of motorcycling), and for consistency with the nonparametric algorithm of the random forest. 

#### 2.9.6. Missing Values

The global sample size was 70 participants. However, due to various obstacles encountered during the experiment (mainly professional availability; hardware malfunctions; inability to record some of the measures for some participants; see explanations above), the sample size for each variable was less than 70 (see Table 1 for details). 

## 3. Results

**Figure 5:**
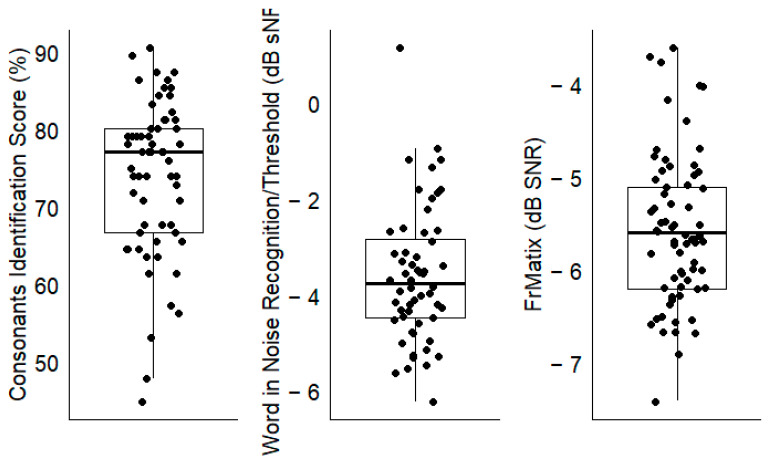
The performance for each speech-in-noise audiometry test; each dot shows the result of one participant. The boxplots show the medial (horizontal bar) and the interquartile range (box). The whiskers reach from the lowest to the highest observed value within 1.5 times the interquartile range.

For the three speech-in-noise audiometry tests, large inter-individual differences were observed, as evidenced by the large interquartile ranges (Figure 5). The results for all the other tests (i.e., the predictors) are represented in Appendix A.

### 3.1. Consonant Identification

#### 3.1.1. Predictor Importance

**Figure 6:**
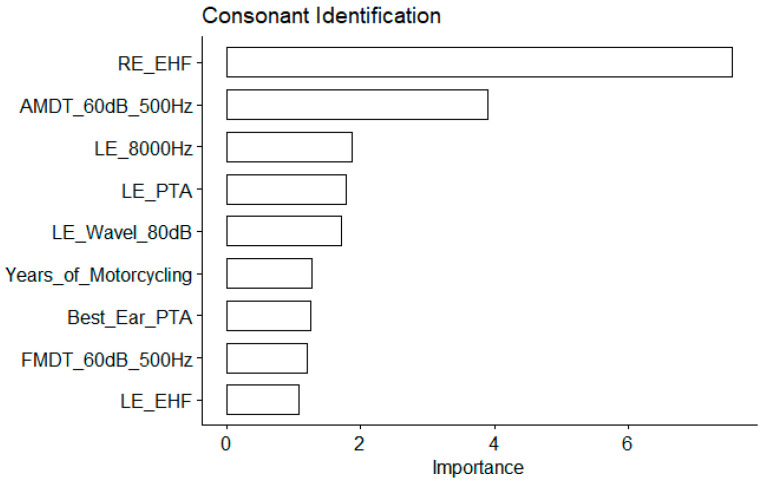
Main predictors of the consonant identification score. The importance is measured as the MSE increase for the nine first most important variables. The larger the value, the more important the variable. See Table 1 for abbreviations.

The EHF hearing threshold was the most important variable by far (an importance value of 7.5 as compared to 3.9 for the AMDT_60 dB_500 Hz; see Figure 6). For the consonant identification scores, the conventional audiometric values turned out to be important predictors too: the 8000 Hz audiometric thresholds; the PTA for the left ear; and the best ear PTA (Figure 6).

#### 3.1.2. Correlations between Predictors and Consonant Identification Scores

**Figure 7:**
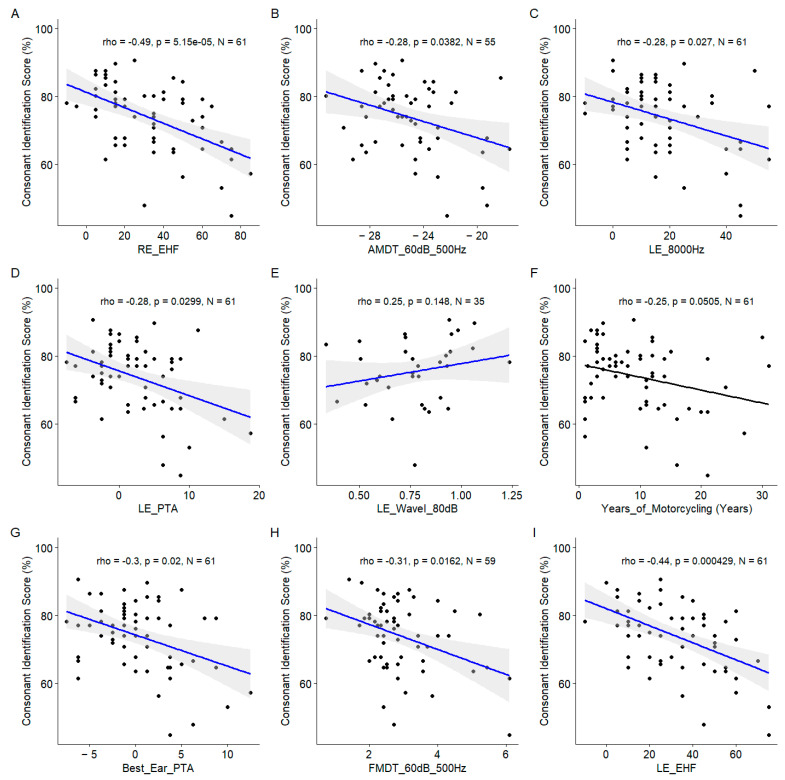
Scatter plots of the nine most important predictors of the consonant identification score. (**A**). Right ear EHF threshold. (**B**). Amplitude modulation detection threshold at 60 dB SL at 500 Hz. (**C**). Left ear 8000Hz threshold. (**D**). Left ear pure tone average. (**E**). Left ear wave I amplitude at 80 dB nHL. (**F**). Years of motorcycling. (**G**). Best ear pure tone average. (**H**). Frequency modulation detection threshold at 60 dB SL at 500 Hz. (**I**). Left ear EHF threshold. In each panel, the Spearman coefficient of correlation, its *p*-value, and the sample size are shown. When the correlation is significant (*p* < 0.05), a blue line indicates a linear fit. The gray region indicates the 95% confidence interval of the regression line.

Significant correlations were also observed for the suprathreshold measures of the temporal coding, namely, the AM detection threshold and the FM detection threshold both measured at 60 dBSL for a 500 Hz carrier frequency (Figure 7). 

### 3.2. Words-in-Noise Recognition

#### 3.2.1. Predictor Importance

**Figure 8:**
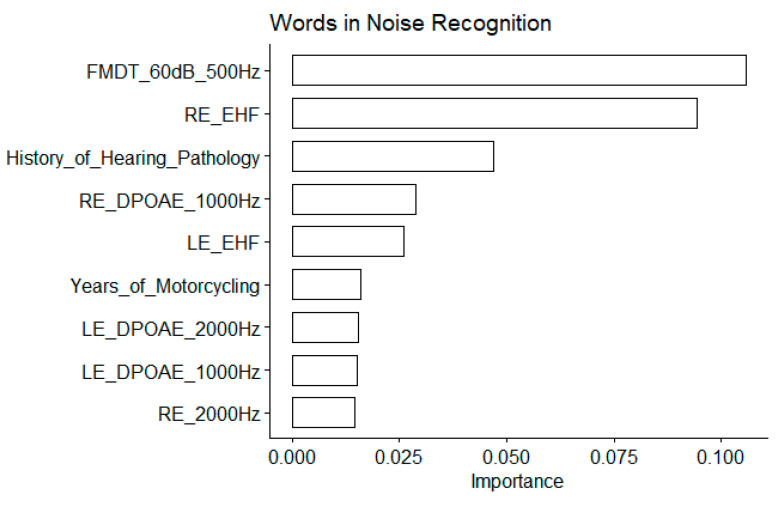
Word in noise recognition. Importance measured as the increase in the mean square error for the nine most important variables. The larger the value is, the more important the variable in the model. See Table 1 for abbreviations.

**Figure 9:**
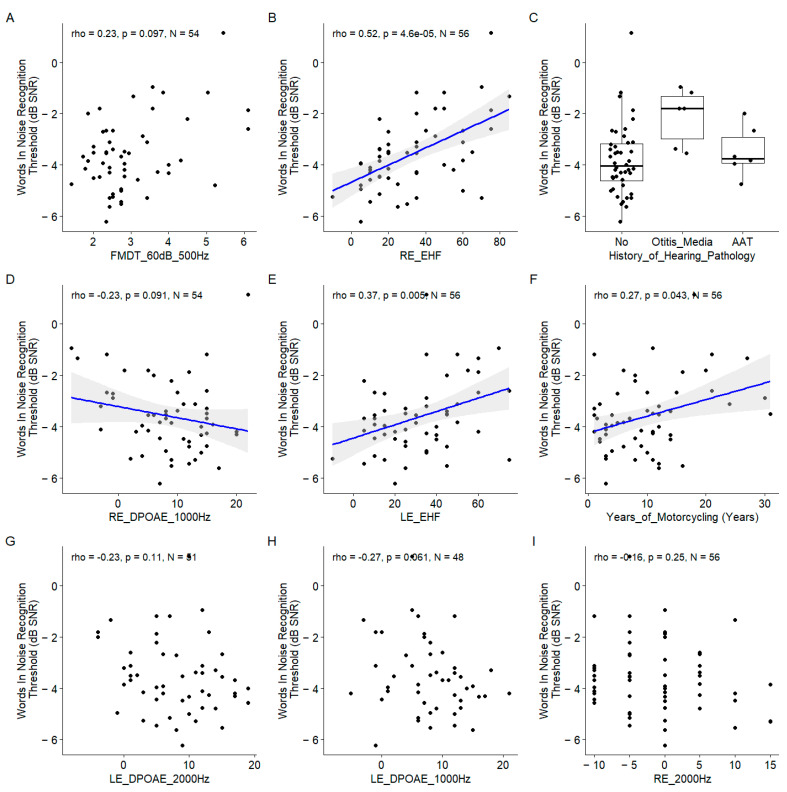
Scatter plots of the nine most important predictors of the word in noise recognition threshold. (**A**). Frequency modulation detection threshold at 60 dB SL at 500 Hz. (**B**). Right ear EHF threshold. (**C**). History of hearing pathology. (**D**). Left ear 1000 Hz threshold. Pure tone average. (**E**). Left ear EHF threshold. (**F**). Years of motorcycling. (**G**). Left ear DPOAE at 2000 Hz. (**H**). Left ear DPOAE at 1000 Hz. (**I**). Left ear 2000 Hz threshold. In each panel, the Spearman coefficient of correlation, its *p*-value, and the sample size are shown. When the correlation is significant (*p* < 0.05), a blue line indicates a linear fit. The gray region indicates the 95% confidence interval of the regression line.

For this speech-in-noise audiometry test, the EHF thresholds at both ears were also among the most important variables, and their correlation with the words-in-noise recognition thresholds were among the highest (Figure 8). The FM detection threshold at 60 dB SL for a 500 Hz carrier frequency appears to be the most important but the correlation with the words-in-noise recognition results was nonsignificant (Figure 9).

#### 3.2.2. Correlations between Predictors and Words-in-Noise Recognition Scores

This discrepancy could be due to the interaction between predictors not assessed here. Similarly, the DPOAEs appeared important but the coefficient of correlation with the words-in-noise recognition results was not significant. The years of motorcycling practice were correlated with the words-in-noise recognition results. The history of hearing pathology was pointed out as an important variable. Indeed, the participants with a history of otitis media had poorer words-in-noise recognition results.

### 3.3. French Matrix Test

#### 3.3.1. Predictor Importance

**Figure 10:**
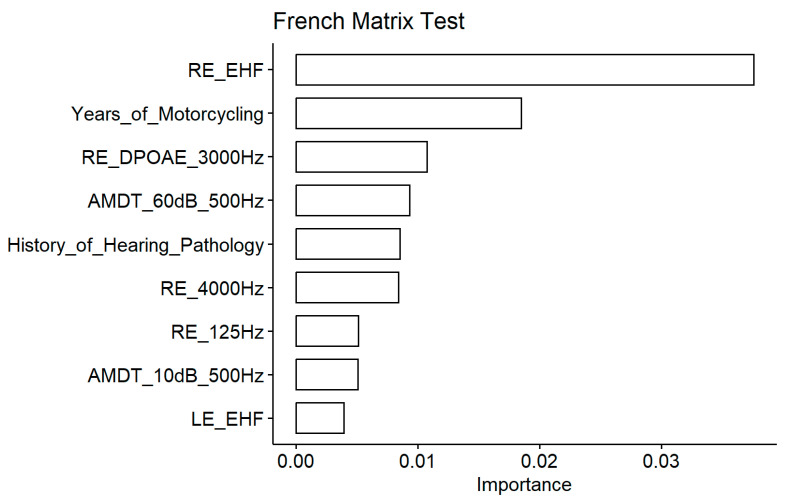
French matrix test. Importance measured as the increase in the mean square error for the nine most important variables. The larger the value is, the more important the variable in the model.

**Figure 11:**
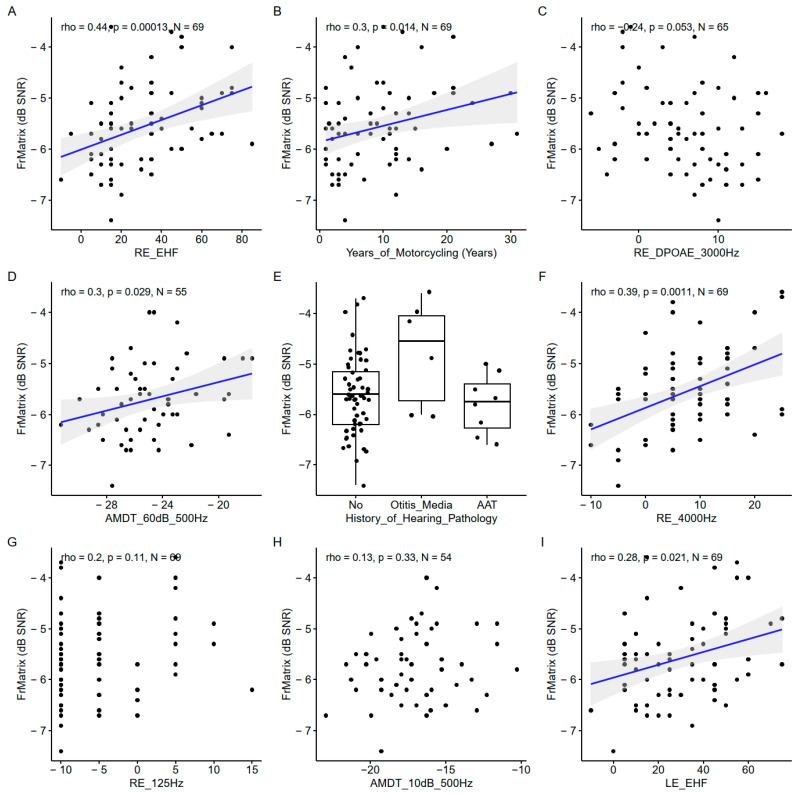
Scatter plots of the nine most important predictors of the FrMatrix. (**A**). Right ear EHF threshold. (**B**). Years of motorcycling. (**C**). Right ear DPOAE at 3000 Hz. (**D**). Amplitude modulation detection threshold at 60 dB SL at 500 Hz. (**E**). History of hearing pathology. (**F**). Right ear 4000 Hz threshold. (**G**). Right ear 125 Hz threshold. (**H**). Amplitude modulation detection threshold at 60 dB SL at 500 Hz. (**I**). Left ear EHF threshold. In each panel, the Spearman coefficient of correlation, its *p*-value, and the sample size are shown. When the correlation is significant (*p* < 0.05), a blue line indicates a linear fit. The gray region indicates the 95% confidence interval of the regression line.

Again, the EHF thresholds were the most important predictors of the FrMatrix score (Figure 10). Two conventional audiometric thresholds were also among the important variables (4000 Hz and 125 Hz measured in the right ear) but only the correlation with the 4000 Hz audiometric threshold was significant (Figure 11). The AM detection threshold with a 500 Hz carrier frequency appeared important but the correlation was significant only at 60 dB SL (like with the consonant identification score) and not at 10 dB SL. 

#### 3.3.2. Correlations between Predictors and French Matrix Test Scores

Like with the words-in-noise recognition results, the FrMatrix was related to the years of motorcycling practice. The DPOAEs appeared among the important variable; nevertheless, the coefficient of correlation did not reach significance. The history of hearing pathology was also an important variable, and, similarly to the words-in-noise recognition, the participants with a history of otitis media showed weaker scores.

### 3.4. Speech-in-Noise Pragmatic Scale from the Speech, Spatial and Quality of Hearing Questionnaire

#### 3.4.1. Predictor Importance

**Figure 12:**
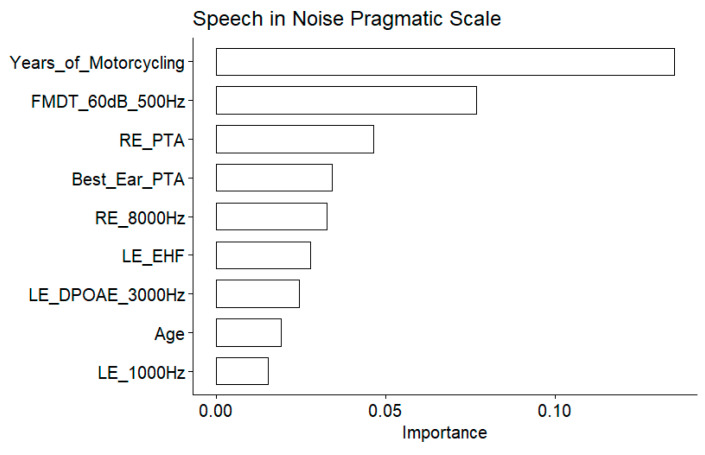
Speech-in-noise pragmatic scale. Importance measured as the increase in the mean square error for the nine most important variables. The larger the value is, the more important the variable in the model.

**Figure 13:**
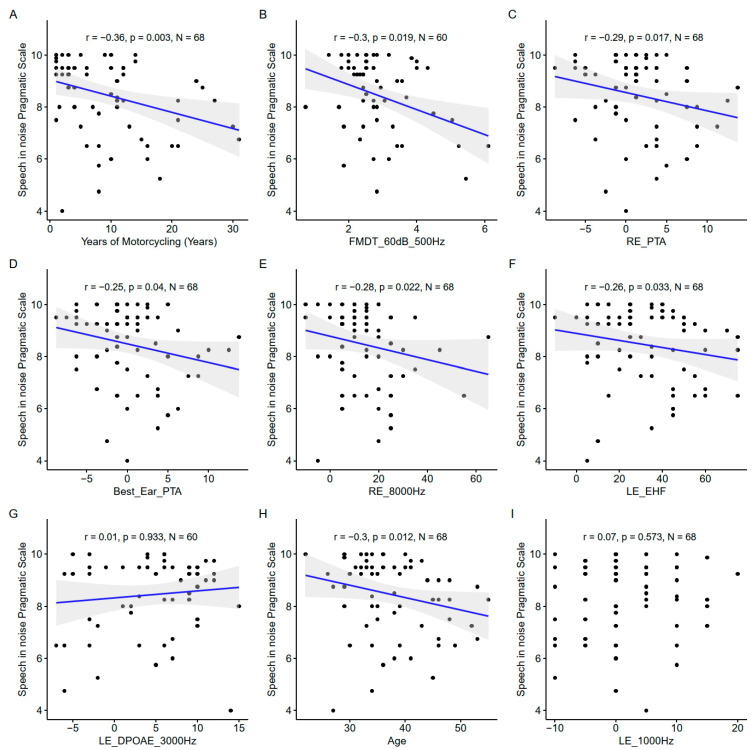
Scatter plots of the nine most important predictors of speech-in-noise pragmatic scale. (**A**). Years of motorcycling. (**B**). Frequency modulation detection threshold at 60 dB SL at 500 Hz. (**C**). Right ear pure tone average. (**D**). Best ear pure tone average. (**E**). Right ear 8000 Hz threshold. (**F**). Left ear EHF threshold. (**G**). Left ear DPOAE at 3000 Hz. (**H**). Age. (**I**). Right ear 1000 Hz threshold. In each panel, the Spearman coefficient of correlation, its *p*-value, and the sample size are shown. When the correlation is significant (*p* < 0.05), a blue line indicates a linear fit. The gray region indicates the 95% confidence interval of the regression line.

The years of motorcycling practice was the most important variable as assessed by the model (Figure 12), and confirmed by the significant correlation with the speech-in-noise pragmatic scale (Figure 13). 

#### 3.4.2. Correlations between Predictors and Speech-in-Noise Pragmatic Scale

The EHF was found to be significantly correlated to the speech-in-noise pragmatic scale. Several conventional audiometric thresholds were also present among the important variables: the right ear PTA, the right ear 8000 Hz threshold, and the left ear 1000 Hz threshold. However, the correlation with the left ear 1000 Hz threshold was not significant. Age also appeared to be important. The DPOAE measured at 3000 Hz in the left ear appeared to be one of the important variables but the correlation was nonsignificant.

### 3.5. Relationship across the Speech-in-Noise Tests

#### 3.5.1. Speech-in-Noise Tests 

**Figure 14:**
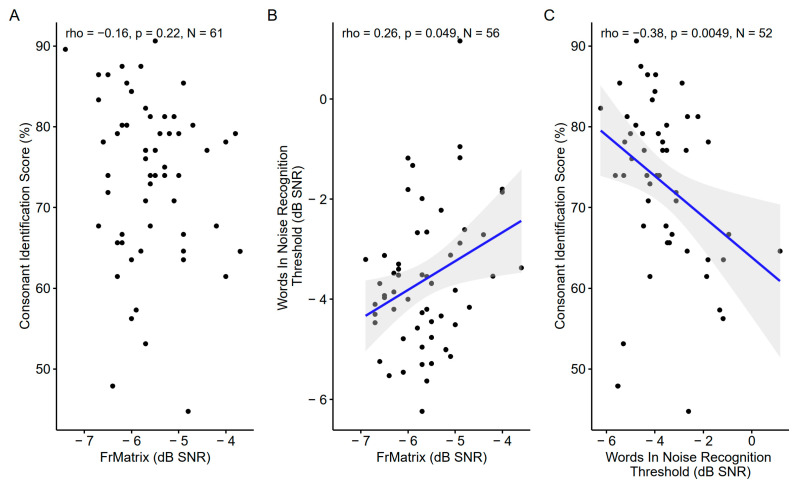
Scatter plots showing the correlations between the three speech-in-noise tests: (**A**). Consonant identification vs. French matrix test. (**B**). Words-in-noise recognition vs. French matrix test. (**C**). Consonant identification vs. words-in-noise recognition. The blue line indicates a linear fit. The gray region indicates the 95% confidence interval of the regression line. In each panel, the Spearman coefficient of correlation, its *p*-value, and the sample size are shown. When the correlation is significant (*p* < 0.05), a blue line indicates a linear fit. The gray region indicates the 95% confidence interval of the regression line.

The consonants in the noise score correlated with the words-in-noise recognition, which was correlated with the FrMatrix (Figure 14). However, the consonants in the noise score were not correlated with the FrMatrix.

#### 3.5.2. Speech-in-Noise Pragmatic Scale and Speech-in-Noise tests 

**Figure 15:**
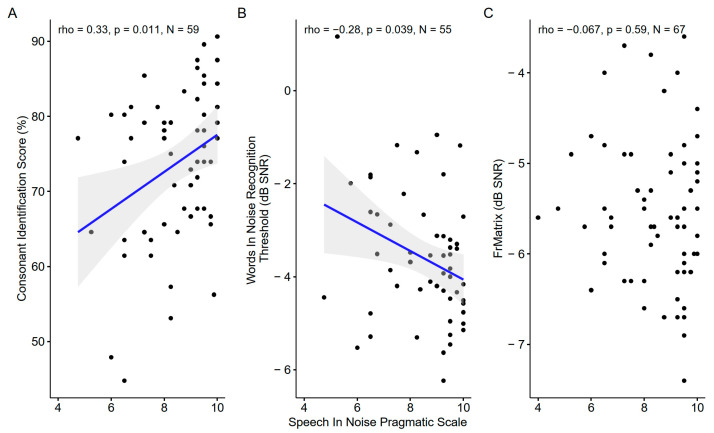
Scatter plots showing the correlations between the speech-in-noise pragmatic scale and the three speech-in-noise tests. (**A**). Consonant identification vs Speech-in-noise pragmatic scale (**B**). Words-in-noise recognition vs. Speech-in-noise pragmatic scale. (**C**). French matrix test vs. Words-in-noise recognition. The blue line indicates a linear fit. The gray region indicates the 95% confidence interval of the regression line. In each panel, the Spearman coefficient of correlation, its *p*-value, and the sample size are shown. When the correlation is significant (*p* < 0.05), a blue line indicates a linear fit. The gray region indicates the 95% confidence interval of the regression line.

The self-reported speech-in-noise pragmatic scale was correlated with the consonant identification score and the words-in-noise recognition threshold but not with the FrMatrix score (Figure 15). 

## 4. Discussion

The aim of this study was to identify the most relevant variables to monitor in a population of noise-exposed professionals. Different physiological and behavioral variables were assessed as predictors of three speech-in-noise tests and the self-reported speech-in-noise abilities. Despite the weak correlations between the speech-in-noise tests, the EHF threshold appeared to be the variable most often related to the speech-in-noise scores. Among the behavioral variables, those related to temporal coding and conventional audiometric thresholds were also highlighted. Concerning the physiological variables, the DPOAE measured at 1000 and 3000 Hz appeared to be the most important variables, although the correlations were weaker than those between the behavioral variables mentioned above.

### 4.1. Comparisons between Speech-in-Noise Tests

To the best of our knowledge, no previous study has looked into the relationship between speech-in-noise audiometry tests with normal- or near-normal-hearing listeners. 

In the current study, weak correlations were observed between the three speech-in-noise audiometry tests. Those weak correlations could illustrate the fact that the speech-in-noise audiometry tests differed in their demand on auditory vs. non-auditory abilities (e.g., linguistic abilities, working memory [12]). Most probably, the consonant identification test has the lowest cognitive demand in comparison to the other tests. This is because of the use of non-word stimuli, which do not require linguistic skills, and the use of a closed-set paradigm that do not demand working memory skills as much [12]. The FrMatrix test probably has the highest cognitive demand with a sentence of five words and an open-set paradigm, although the number of alternatives per word categories is limited to ten. The words-in-noise recognition test is assumed to have intermediate cognitive demand as it was designed to reduce cognitive demand by choosing frequent French words with phonological neighbors, while presented in an open-set paradigm [44]. The hierarchy in cognitive demand across the three speech-in-noise audiometry tests could explain the pattern of observed correlation across the tests: consonant identification was correlated only with words-in-noise recognition, while the FrMatrix was correlated only with the words-in-noise recognition. This result emphasizes, within the same group of participants, the relative influence of sensory and cognitive factors on speech-in-noise task performance [12]. This is coherent with the results reviewed by Dryden et al. [11]. However, intra-individual variability due to learning effects [9] could have blurred the correlations across the tests. This learning effect bias was controlled firstly by conducting three FrMatrix sessions, and secondly by providing a closed-set response choice in the consonant identification test. Finally, although the learning effect cannot be excluded in the words-in-noise recognition test, that test was designed to reduce top-down influences [45].

Moreover, the three speech-in-noise audiometry tests assessed in the current study also presented weak correlations with the speech-in-noise pragmatic scale of the SSQ, a self-reported measure. This could emphasize the importance of using tests other than questionnaires in a prevention program. However, the lack of a relationship could also reflect the low ecological aspect of the tests. None of the speech-in-noise tests used in the current study relied on semantic context or everyday sentences. In a large sample study (N = 195 near-normal-hearing listeners), Stenbäck and colleagues [46] found a correlation between the speech SSQ scale and a speech-in-noise test using everyday sentences (HINT [47]), but not with a less ecologically valid test [48]. This point will still need to be reproduced and confirmed before being implemented in clinical prevention programs.

### 4.2. Speech-in-Noise Test Predictors

#### 4.2.1. Audiometric Thresholds

The importance of the EHF thresholds as a predictor for the three speech-in-noise audiometry tests is one of the most important results here. 

Correlations between the EHF threshold and speech-in-noise tests have been highlighted in many previous studies involving normal-hearing listeners [29], including populations similar to ours (middle aged, near-normal hearing thresholds [32,49]. Two explanations, at least, have been suggested [18,49]. The first explanation is a direct causal relationship: the speech signal in the frequency region above 8 kHz could be useful to identify words presented in noisy background [50], especially consonants, like voiceless fricatives [51]. We have retrospectively explored this hypothesis by analyzing the signal spectrum of the speech from the three tests used here. Almost all of the acoustic energy was below 8 kHz (see results in Appendix B), suggesting that this explanation is not adapted to our study. 

A second possible explanation is that chronic exposure to high-level noise could cause both cochlear synaptopathy, which alters speech-in-noise performance, and damage to basal outer hair cells, which causes EHF threshold shift [29]. Indeed, extreme basal outer hair cells do not stand the oxidative stress produced by their overstimulation due to high noise levels because of their incapacity to maintain calcium homeostasis [52]. In the same way, high noise levels induce the massive and toxic release of glutamate in the synapses of low-spontaneous-rate auditory fibers, leading to the destruction of the synapses [53]. 

A third explanation could be that the tuning curve of auditory fibers coding for EHF broadens when the acoustic stimulation reaches high levels. Thus, they could provide temporal information and improve the coding of lower frequencies as well [50]. Nevertheless, in our study, this explanation is unlikely given the relatively moderate levels used for stimulus presentation (around 65 dB SPL). 

In addition, the EHF threshold was found to be correlated with several different cochlear aggressors beyond those suspected of inducing synaptopathy (age and noise), but also drugs (cisplatin), diabetes [54], or smoking [55]. Therefore, the EHF threshold could be a kind of cochlear aggressor integrator, with each aggressor affecting speech understanding in various ways.

The high-frequency conventional audiometric thresholds and/or their combination represent several important variables for the consonant identification and FrMatrix tests. These results show that the audiometric thresholds remain informative for normal- or near-normal-hearing listeners. Most of the studies involving normal-hearing listeners explained individual differences in the speech-in-noise performance by noise-induced synaptopathy, even if the mere existence of noise-induced cochlear synaptopathy in living humans is still questionable [25]. Our results suggest that individual differences in conventional audiometric thresholds (EHF excluded), even if they remain in the “normal” range, are also interesting to explain individual differences in the speech-in-noise performance for a noise-exposed population. Not dividing our participants in several groups, or comparing them with another non-exposed control group, was one of the key points in our study design. Indeed, it is interesting to note here that conducting a study based on group comparisons, when the individuals within the group are very homogeneous in terms of conventional audiometric thresholds, might not be a relevant strategy to evaluate the predictors of speech-in-noise performance. Nevertheless, this strategy is often used to compare groups according to their noise exposure to search for noise-induced synaptopathy [20,27,29].

Finally, we think that focusing on a group of individuals exposed to one type of noise (motorcycle) in a very similar way (with one main variable link to the number of years of motorcycling) was instrumental to limit the large variability inherent to this type of study. The motorcycle noise could have caused both synaptopathy and audiometric threshold shift through outer hair damage, gradually and similarly among the population. In other studies exploring populations with normal hearing but different kinds of noise exposure, the correlation could have been blurred: some types of noise could have a greater impact on outer hair cells (impulse noise) and other types of noise cause more impact on the synapses (steady state noise) [26].

#### 4.2.2. Amplitude and Frequency Modulation Detection

High permutation importance and significant correlations were observed between the consonant identification score and the AM and FM detection thresholds for low-frequency carrier and high-intensity level. Measures of temporal coding also appeared to be important for the words-in-noise recognition and the FrMatrix.

These results are consistent with an alteration in the temporal coding abilities induced by the synaptopathy, as has been suggested in humans [56] and in rodents [57]. The synaptopathy reduces the number of auditory fibers, hence the fidelity of the neural phase locking. Indeed, to obtain usable information, the activity of several fibers must be combined, due to their stochastic activity. However, temporal coding is thought to play a role in speech comprehension in noisy environments [17,58], by helping the segregation between the noise and signal. Moreover, as temporal coding is crucial for segregation between speech streams, a higher importance of variables related to temporal coding would have been expected for speech-in-speech tasks [17] rather than speech-in-noise tasks, as used in the current study. Furthermore, in our middle-aged population, aging per se [59] or age-related synaptopathy [60] could have also altered the temporal coding abilities of the participants.

#### 4.2.3. Age

Age did not appear to be one of the important variables for any of the speech-in-noise audiometry tests. This was an unexpected result given that, even in the age span of our population, aging can alter the speech-in-noise performance in many ways (alteration of outer hair cells, of synapses between inner hair cells and auditory fibers [60], decrease in temporal coding abilities) [59,61]. The fact that age did not appear as a factor per se could mean that its effect was well captured by the measurements as well as the variable “years of motorcycling”. However, age is also related to a factor that we did not explore in our study: the working memory. The working memory is expected to play a role in the speech-in-noise intelligibility according to recent models (cognitive hearing science) [62,63]. The working memory could be altered even within our population’s age span [64]. Nevertheless, the working memory was perhaps not relevant in our study for explaining the individual differences in the speech-in-noise performance. First, a meta-analysis showed that the working memory was not a relevant factor to explain the individual differences among normal-hearing listeners [65]. Second, although we did not explicitly measure it, the level of education, which is related to the working memory, was relatively homogeneous in our population. Moreover, in our study, we used only speech-in-noise tasks. A speech-in-speech task would have shown a stronger relationship with age [66].

#### 4.2.4. Physiological Measurements: Distortion Products of Otoacoustic Emissions and Electrocochleography

The amplitude of wave I was not related to the speech-in-noise performance in our study. However, we hypothesized that their exposure to noise should have caused synaptopathy. By definition, synaptopathy implies a decrease in the number of synapses, hence, a lower amplitude of wave I. However, many studies did not observe a correlation between the wave I amplitude and speech-in-noise performance [25,27], in contrast to the findings of [29,67]. Methodological aspects have been proposed to explain this discrepancy [25]. The main difficulty is to target the low-spontaneous-rate fibers [53] in the recorded electrical signal. Different strategies have been used (wave I growth function [68], summing potential/action potential ratio [29]). A promising technique would be to isolate the low-spontaneous-rate fibers thanks to an ipsilateral noise saturating the high-spontaneous-rate fibers [69]. In our study, the three tests relied on energetic masking. Nevertheless, informational masking with different intelligible speech streams would perhaps have been more efficient to reveal the relationships with EcochG [12]. Indeed, the segregation of speech streams requires sufficient temporal coding abilities to identify the target speech based on voice cues or localization cues. As the temporal coding of speech signals is supposed to be strongly linked to the number of functional low-spontaneous-rate fibers, synaptopathy would be particularly efficient to reduce speech-in-speech intelligibility. 

Previous studies have suggested that the outer hair cell function could also be responsible for speech-in-noise performance [22]. Our results confirmed this by highlighting the DPOAE as an important predictor for words-in-noise recognition and the FrMatrix. Interestingly, Parker [22] found correlations between the speech-in-noise tests and the DPOAE in the same range of frequencies and magnitude of correlation coefficients but for a lower intensity of stimulation (65/55 vs. 75/65 dB SPL). Our parameters of the DPOAE intensity stimulation were not classical [70]. Nevertheless, they probably helped improve the signal-to-noise ratio.

### 4.3. Clinical Implications

The importance of EHF thresholds to predict the speech-in-noise performance suggests that it could be a relevant variable for screening a noise-exposed population. With most commercial audiometers, it is now possible to measure the EHF thresholds with the same equipment as the conventional frequencies, at least up to 12,500 Hz. If an EHF threshold shift is detected, then further tests could be proposed in order to target the auditory dysfunction in more detail. Tests could include speech-in-noise audiometry, physiological measurements (DPOAE, EcochG), and/or questionnaires (SSQ, tinnitus screening questionnaire). Finally, the early diagnosis of an auditory dysfunction would lead to appropriate and timely medical care (hearing aids, strategies for challenging listening situations, strategies to protect residual hearing) and prevent accompanying disabilities. Therefore, we suggest that EHF testing should be performed as a new standard [18,71]. 

### 4.4. Limits of the Study

The interpretation of the results was complicated by the fact that some variables were identified as important although non significantly related to the speech-in-noise performance. That result could indicate potential complex interactions between the variables, which could be explored in further studies employing a larger sample of participants. Another limitation of the study is the lack of gender diversity, which is, unfortunately, inherent to the studied population. It represents a side effect of choosing a homogeneous exposed population. Another side effect is the potential lack of the generalizability of the results to the entire population. Future works could be conducted with groups of subjects exposed to different kinds of occupational noise.

## 5. Conclusions

A high number of behavioral or physiological measures are related to speech intelligibility in noisy backgrounds. The EHF threshold appears to be one of the most important factors for predicting different speech-in-noise tests within a noise-exposed population. The EHF threshold could reveal not only the impact of noise exposure but also of numerous auditory aggressors. Therefore, adding EHF thresholds to the monitoring of a noise-exposed population could help to prevent difficulties in understanding speech in noise and an alteration of their professional and personal lives.

## Figures and Tables

**Table 1:** Sample size of each combination of variable and conditions of the dataset. AMDT: detection threshold of AM, FMDT: detection threshold of FM, DPOAE: distortion products of otoacoustic emission.

Test	Conditions	N	Abbreviation
Consonant identification		61	
Word in noise recognition		56	
French matrix test		69	FrMatrix
Age		70	Age
History of hearing pathology		70	History_of_Hearing_Pathology
Years of motorcycling		70	Years_of_Motocycling
AMDT	60 dB SL 4000 Hz	42	AMDT_60 dB_4000 Hz
	60 dB SL 500 Hz	55	AMDT_60 dB_500 Hz
	10 dB SL 4000 Hz	55	AMDT_10 dB_4000 Hz
	10 dB SL 500 Hz	55	AMDT_10 dB_500 Hz
FMDT	60 dB SL 4000 Hz	22	FMDT_60 dB_4000 Hz
	60 dB SL 500 Hz	61	FMDT_60 dB_500 Hz
	10 dB SL 4000 Hz	23	FMDT_10 dB_4000 Hz
	10 dB SL 500 Hz	45	FMDT_10 dB_500 Hz
	60 dB SL 4000 Hz Ability	60	FMDT_60 dB_4000 Hz_Ab
	10 dB SL 4000 Hz Ability	61	FMDT_10 dB_4000 Hz_Ab
	10 dB SL 500 Hz Ability	61	FMDT_10 dB_500 Hz_Ab
DPOAE	Left Ear 1000 Hz	59	LE_DPOAE_1000 Hz
	Left Ear 1500 Hz	62	LE_DPOAE_1500 Hz
	Left Ear 2000 Hz	62	LE_DPOAE_2000 Hz
	Left Ear 3000 Hz	62	LE_DPOAE_3000 Hz
	Left Ear 4000 Hz	62	LE_DPOAE_4000 Hz
	Left Ear 5000 Hz	58	LE_DPOAE_5000 Hz
	Right Ear 1000 Hz	65	RE_DPOAE_1000 Hz
	Right Ear 1500 Hz	63	RE_DPOAE_1500 Hz
	Right Ear 2000 Hz	65	RE_DPOAE_2000 Hz
	Right Ear 3000 Hz	65	RE_DPOAE_3000 Hz
	Right Ear 4000 Hz	65	RE_DPOAE_4000 Hz
	Right Ear 5000 Hz	61	RE_DPOAE_5000 Hz
Tonal audiometry	Left Ear 125 Hz	70	LE_125 Hz
	Left Ear 250 Hz	70	LE_250 Hz
	Left Ear 500 Hz	70	LE_500 Hz
	Left Ear 1000 Hz	70	LE_1000 Hz
	Left Ear 2000 Hz	70	LE_2000 Hz
	Left Ear 4000 Hz	70	LE_4000 Hz
	Left Ear 8000 Hz	70	LE_8000 Hz
	Left Ear EHF	70	LE_EHF
	Left Ear PTA	70	LE_PTA
	Right Ear 125 Hz	70	RE_125 Hz
	Right Ear 250 Hz	70	RE_250 Hz
	Right Ear 500 Hz	70	RE_500 Hz
	Right Ear 1000 Hz	70	RE_1000 Hz
	Right Ear 2000 Hz	70	RE_2000 Hz
	Right Ear 4000 Hz	70	RE_4000 Hz
	Right Ear 8000 Hz	70	RE_8000 Hz
	Right Ear EHF	70	RE_EHF
	Right Ear PTA	70	RE_PTA
	Best Ear PTA	70	Best_Ear_PTA
Electrocochleography	Left Ear Wave I 80 dB HL	37	LE_WaveI_80 dB
	Left Ear Wave I 90 dB HL	38	LE_WaveI_90 dB
	Right Ear Wave I 80 dB HL	49	RE_WaveI_80 dB
	Right Ear Wave I 90 dB HL	52	LE_WaveI_90 dB
	Left Ear Wave I Slope	34	LE_Slope
	Right Ear Wave I Slope	46	RE_Slope

**Figure A1:**
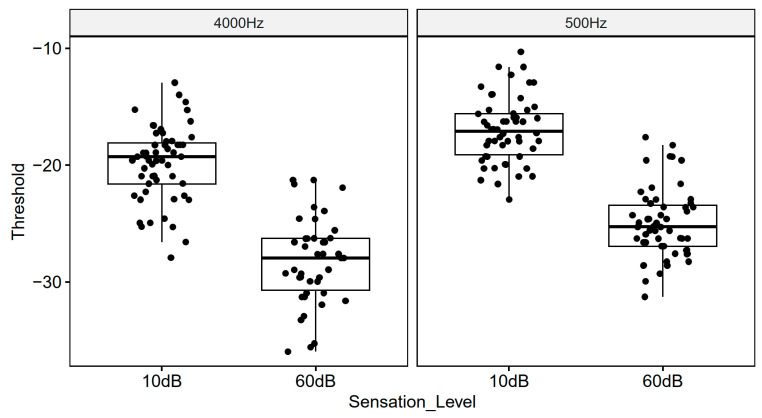
Amplitude modulation detection threshold as a function of sensation level and carrier frequency. The boxplots show the medial (horizontal bar) and the interquartile range (box). The whiskers reach from the lowest to the highest observed value within 1.5 times the interquartile range. Dots show individual points.

**Figure A2:**
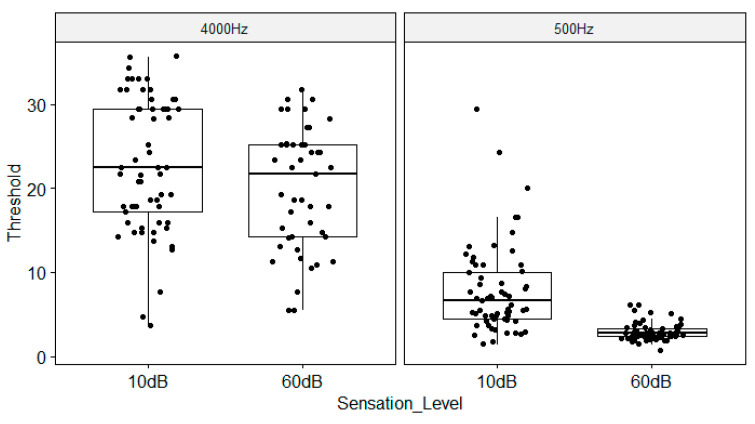
Frequency modulation detection threshold as a function of sensation level and carrier frequency. The boxplots show the medial (horizontal bar) and the interquartile range (box). The whiskers reach from the lowest to the highest observed value within 1.5 times the interquartile range. Dots show individual points.

**Figure A3:**
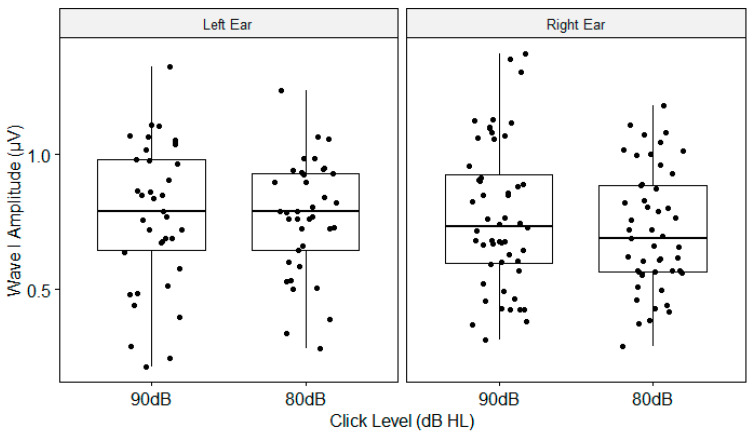
Wave I amplitude as a function of click level and ear. The boxplots show the medial (horizontal bar) and the interquartile range (box). The whiskers reach from the lowest to the highest observed value within 1.5 times the interquartile range. Dots show individual points.

**Figure A4:**
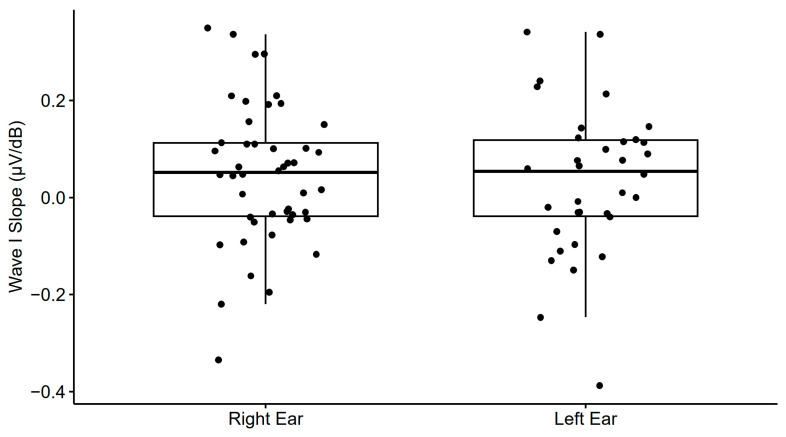
Electrocochleography wave I slope as a function of the ear. The boxplots show the medial (horizontal bar) and the interquartile range (box). The whiskers reach from the lowest to the highest observed value within 1.5 times the interquartile range. Dots show individual points.

**Figure A5:**
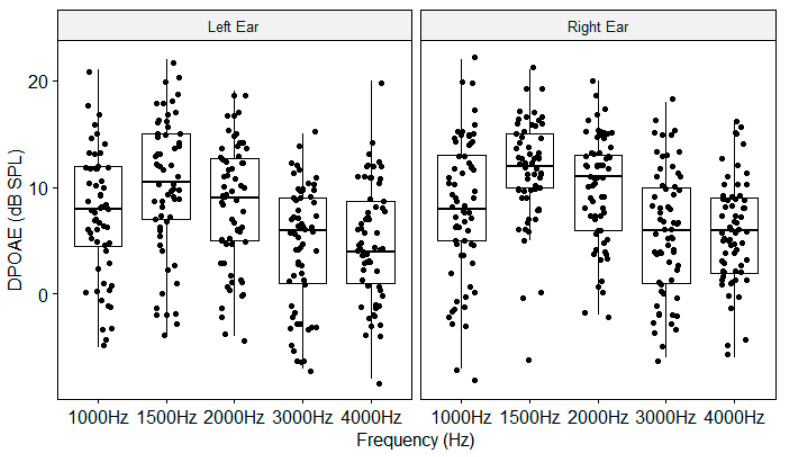
DPOAE as a function of frequency and ear. The boxplots show the medial (horizontal bar) and the interquartile range (box). The whiskers reach from the lowest to the highest observed value within 1.5 times the interquartile range. Dots show individual points.

**Figure A6:**
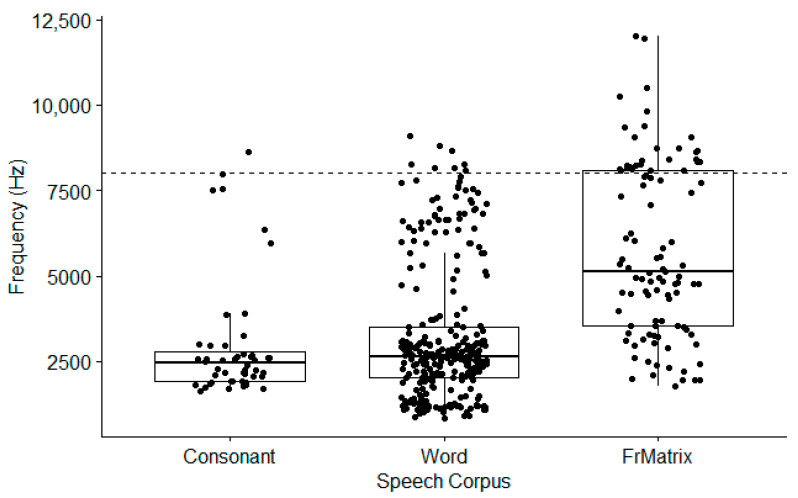
Upper frequency bounds under which 99% of the total power of the spectrum of the speech signals are comprised, for the three speech corpora. The boxplots show the medial (horizontal bar) and the interquartile range (box). The whiskers reach from the lowest to the highest observed value within 1.5 times the interquartile range. Dots show individual speech sounds. The dashed line points out the frequency of 8000 Hz.

**Figure A7:**
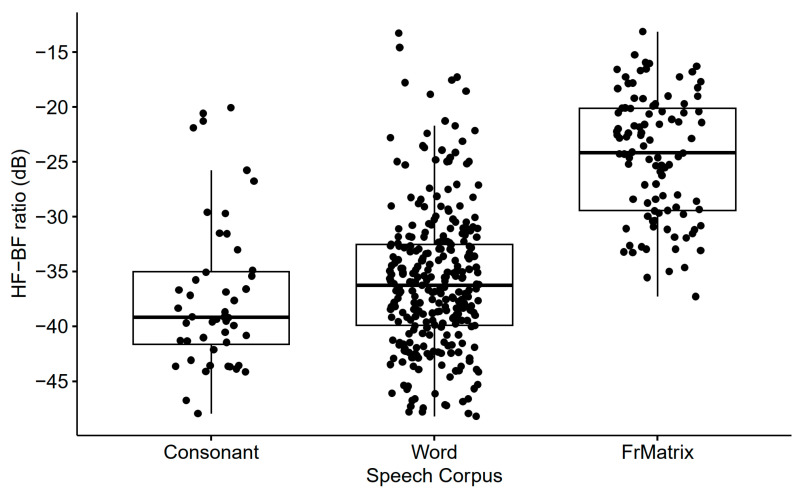
Ratio of acoustical power contained in high vs. in low frequencies, with a cutoff frequency of 8 kHz, for the three speech corpora. The boxplots show the medial (horizontal bar) and the interquartile range (box). The whiskers reach from the lowest to the highest observed value within 1.5 times the interquartile range. Dots show individual speech sounds. The majority of speech sounds present at least 20 dB more energy in low (below 8 kHz) vs. in high frequencies.

## Data Availability

The raw data supporting the conclusions of this article will be made available by the authors on request.

## Appendix B

### Appendix B.1. Acoustical Analyses of the Three Speech Corpora

For these analyses, the ‘consonant’ and ‘words’ corpora contain all 48 and 291 sound files, respectively, while the ‘FrMatrix’ corpus contains the recordings of 100 random sentences.
